# Greater Risk of Periprosthetic Joint Infection Associated with Prolonged Operative Time in Primary Total Knee Arthroplasty: Meta-Analysis of 427,361 Patients

**DOI:** 10.3390/jcm13113046

**Published:** 2024-05-22

**Authors:** Kyun-Ho Shin, Jin-Ho Kim, Seung-Beom Han

**Affiliations:** 1 Department of Orthopedic Surgery, Yeson Hospital, Bucheon 14555, Republic of Korea; jhkayo@naver.com; 2 Department of Orthopedic Surgery, Anam Hospital, Korea University College of Medicine, Seoul 02841, Republic of Korea; oahan@korea.ac.kr

**Keywords:** total knee arthroplasty, prosthesis-related infections, surgical wound infection, operative time, periprosthetic joint infection

## Abstract

**Background/Objectives:** Periprosthetic joint infection (PJI) is a severe complication in total knee arthroplasty (TKA) with catastrophic outcomes. The relationship between prolonged operative times and PJI remains debated. This meta-analysis investigated the link between prolonged operative times and the risk of PJI in primary TKA. **Methods:** A comprehensive search of the MEDLINE/PubMed, Cochrane Library, and EMBASE databases was conducted to identify studies comparing the incidence of PJI in TKAs with prolonged versus short operative times, as well as those comparing operative times in TKAs with and without PJI. Pooled standardized mean differences (SMD) in operative times between groups with and without PJI or surgical site infections (SSI), including superficial SSIs and PJIs, were analyzed. Additionally, the pooled odds ratios (OR) for PJI in TKAs with operative times exceeding 90 or 120 min were examined. **Results:** Seventeen studies involving 427,361 patients were included. Significant differences in pooled mean operative times between the infected and non-infected TKA groups were observed (PJI, pooled SMD = 0.38, *p* < 0.01; SSI, pooled SMD = 0.72, *p* < 0.01). A higher risk of PJI was noted in surgeries lasting over 90 or 120 min compared to those of shorter duration (90 min, pooled OR = 1.50, *p* < 0.01; 120 min, pooled OR = 1.56, *p* < 0.01). **Conclusions:** An association between prolonged operative time and increased risk of PJI in primary TKA has been established. Strategies for infection prevention should encompass thorough preoperative planning aimed at minimizing factors that contribute to prolonged operative times.

## 1. Introduction

Periprosthetic joint infection (PJI) stands as one of the most severe complications of primary total knee arthroplasty (TKA), often resulting in catastrophic consequences [1,2,3,4,5]. Established risk factors for PJI include elevated body mass index, diabetes mellitus, urinary tract infections, allogenic blood transfusion, and rheumatoid arthritis [4,5,6,7,8]. However, these factors are largely non-modifiable, particularly in patients with severe arthritis, for whom surgery should not be delayed.

In recent decades, increasing evidence has suggested that operative time is an independent risk factor of surgical site infection (SSI) in various surgical procedures [9]. From a surgeon’s point of view, operative time is a potentially modifiable factor, in contrast to patient-related factors. However, in the TKA population, previous studies on operative time as a risk factor of PJI have reported conflicting results. Several studies have demonstrated that longer surgeries are associated with increased risk for infection [10,11,12,13,14,15,16], whereas other studies have failed to report such an association [17,18,19,20,21,22].

To the best of our knowledge, no previous study has conducted a comprehensive review to assess and quantify the association between operative time and PJI in primary TKA. Given the impact of PJIs on patient outcomes and healthcare burdens, the aim of this meta-analysis was to systematically synthesize the relevant literature that reported on the association between operative time and PJI and quantify the magnitude of the risk of prolonged operative time in patients undergoing primary TKA. We hypothesized that prolonged operative time would be associated with a greater risk of developing PJI following primary TKA. This study underscores the critical importance of managing operative duration in TKA procedures, offering clear guidelines that may help reduce the incidence of PJI, thereby enhancing patient recovery and optimizing resource utilization in clinical settings.

## 2. Materials and Methods

### 2.1. Search Strategy

This meta-analysis followed the Preferred Reporting Items for Systematic Reviews and Meta-Analyses (PRISMA) guidelines [23]. This study is registered with the ResearchRegistry, and the unique identifying number is reviewregistry1823.

We performed an electronic literature search of three online databases, namely, Medline/PubMed, the Cochrane Central Register of Controlled Trials, and EMBASE. The last electronic search was performed on 30 July 2023. No restrictions were applied, including the publication language, study period, or sample size. We entered the following Medical Subject Headings (MeSH) terms and key terms in all fields of the search engines: “total knee replacement” OR “ total knee arthroplasty” OR “arthroplasty, replacement, knee” [MeSH term] AND “infection” AND “Operative Time” [MeSH term] OR “operative time” OR “operating time” OR “operating times” OR “operating room time” OR “operating room times” OR “surgery time” OR “surgery times” OR “surgical time” OR “surgical times” OR “surgical duration” OR “operation duration” OR “operative duration” OR “surgery duration” or “duration of surgery” or “duration of operation” or “time of operation” or “time of surgery.” Following the initial electronic search, the relevant articles and their references were manually searched to identify other suitable articles that were not identified during the database search.

### 2.2. Eligibility Criteria and Study Selection

We selected studies that were eligible on the basis of the following predefined criteria: (1) studies that reported the outcomes in a cohort of patients who underwent primary TKA and excluded those who underwent revisional TKA; (2) studies that evaluated SSI or PJI and did not include generalized infections such as sepsis, urinary tract infection, or pneumonia; and (3) studies in which the outcomes were based on comparisons of operative times between infected and non-infected patients or on comparisons of infection risks between TKAs with or without prolonged operative times, defined as a cutoff value of 90 min and 120 min, as suggested by the Centers for Disease Control and Prevention to identify post-TKA patients at increased risk of infection [24]. The articles were reviewed by two independent reviewers. During the first stage of screening, the reviewers manually checked the titles and abstracts of all relevant articles. The full texts of the articles were reviewed in the second stage of the screening process to select articles that met the inclusion criteria.

### 2.3. Data Extraction

Data were extracted according to the following descriptive information provided in the included trials: (1) study characteristics, including the author names, year of publication, study design, level of evidence, and journal title; (2) composition of the study cohort; (3) definition of infection; (4) definition of operative time; (5) controlled variables other than operative times; and (6) follow-up period. In the case of disagreement between the reviewers with respect to the data collected, the extracted data were subsequently cross-checked for accuracy.

### 2.4. Quality Assessment

Each of the selected studies was evaluated by two independent authors for methodological quality, first independently, and then by consensus. The Newcastle–Ottawa assessment scale was used to assess the methodological quality of the case–control studies [25,26]. The Newcastle–Ottawa assessment scale comprises selection (four categories), comparability, (one category), and outcome domains (three categories). A maximum of one star was assigned for each category within the selection and outcome domains, and a maximum of two stars was given for comparability. Studies with scores ≥ 7 were considered to have a low risk of bias; those with scores 4–6 were considered to have a moderate bias risk; and those with scores 4 were considered to have a high bias risk.

### 2.5. Definition and Outcomes of Interest

Various definitions of SSI and PJI following TKA are available. The Centers for Disease Control and Prevention (CDC) groups SSIs developed within 90 days of the index procedure into superficial (involvement of skin and subcutaneous tissue of the incision) and deep (involvement of fascial and muscle layers of the incision). Deep SSIs are grouped together as deep SSIs and constitute PJI in the context of hip and knee arthroplasty [24]. Additionally, the Musculoskeletal Infection Society (MSIS) workgroup defined algorithmic criteria for the diagnosis of PJI following TKA [27]. In the clinical practice of arthroplasty, surgical infections are generally divided into superficial SSI or deep SSI as PJI [28]. Reoperation, including debridement and removal or exchange of prostheses, is required for the treatment of PJI, while superficial subcutaneous SSI can be treated with antibiotics and incisional drainage if needed. Therefore, in the present study, PJI was defined as either a deep SSI according to the CDC criteria [24], a PJI diagnosed by the MSIS criteria [27], or a deep infection requiring reoperation after TKA. Furthermore, SSI was defined as all infections around the surgical site, including both superficial SSI and PJI.

Two primary outcomes were evaluated: the operative time in infected versus non-infected TKA cases and the incidence rates of PJI at the latest follow-up, with cohorts divided according to operative time cutoffs of 90 and 120 min.

### 2.6. Statistical Analysis

All data from the included studies were extracted into an Excel spreadsheet (Version number 1808, Microsoft Corporation, Redmond, WA, USA). Statistical analyses were performed using the packages meta (v4.17-0) in R Studio statistics program (v.1.4.1106) [29]. A *p*-value < 0.05 was set as the threshold for statistical significance. The operative times were statistically compared between the infected and non-infected groups. The data were standardized for intergroup comparisons of the outcomes because the materials and methods used in the included studies were heterogeneous, such as the definition of operative time. The SMD was defined as the difference in mean outcome divided by the standard deviation of the difference in outcome. The SMD and associated 95% CIs were determined for the operative times. Furthermore, the incidence rates of PJI were compared between a group with prolonged operative times and a control group (patients with short operative times respective to the cutoff value). ORs and 95% CIs were calculated as summary statistics for the incidence rate of PJI.

*I*^2^ statistics were calculated to present the percentage of the total variation attributable to the heterogeneity among the included studies. If there was no heterogeneity (*I*^2^ ≤ 50%), the fixed-effects model was used to merge the effect sizes. If there was heterogeneity (*I*^2^ > 50%), the random-effects model was used to merge the effect sizes [30]. A Sensitivity analysis was conducted to determine the influence of an individual study on the overall pooled effects using the leave-one-out analysis. Publication bias was investigated by evaluating the funnel plot asymmetry and by using an Egger test [31,32]. Forest plots were used to graphically present the results of individual studies and the respective pooled estimate of the effect size.

## 3. Results

### 3.1. Study Selection and Quality Assessment

**Figure 1:**
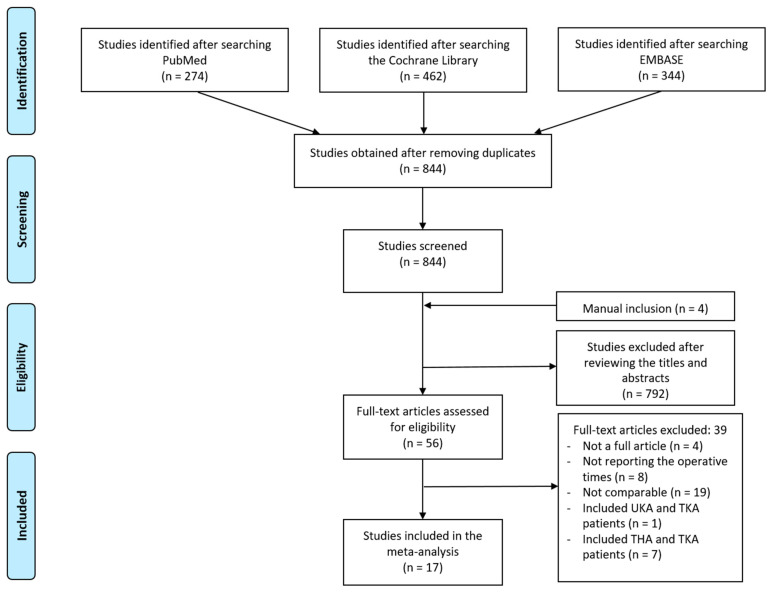
PRISMA flow diagram. PRISMA: Preferred Reporting Items for Systematic Reviews and Meta-Analyses.

Figure 1 shows the process of study identification, inclusion, and exclusion. Electronic searches of the PubMed (Medline), EMBASE, and Cochrane Library databases yielded 274, 462, and 344 studies, respectively. After removing 236 duplicate studies, we obtained 844 studies. Four additional publications were identified through manual searching, among which, 792 were further excluded after reading their abstracts and titles. The full texts of 56 studies were reviewed, and 17 studies were finally included in the meta-analysis after applying the inclusion criteria [10,12,15,16,17,19,20,21,22,32,33,34,35,36,37,38,39]. The main characteristics of the 17 individual studies are summarized in Table 1. Ten studies [10,15,16,17,21,33,34,37,38,40] showed a low risk of bias. The others [12,19,20,22,35,36,39] did not include a description of the adequacy of patient follow-up and showed a moderate risk of bias (Table 2).

### 3.2. Operative Times in the Primary TKA Cases with and without SSI

**Figure 2:**
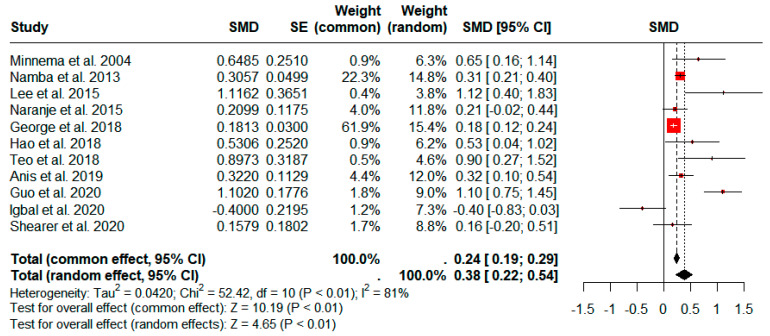
Forest plots showing the operative times of patients with and without surgical site infection. CI, confidence interval; SE, standard error; SMD, standardized mean difference.

Twelve studies comprising 227,547 patients (1905 SSIs) compared the operative times between patients with and without SSI, including both superficial SSI and PJI after TKA [10,12,17,19,20,21,32,35,36,37,38,39]. The SSI group demonstrated a significantly longer operative time based on our pooled analyses using a random-effects model (pooled standardized mean difference (SMD): 0.38; 95% confidence interval (CI): 0.22–0.53; *p* < 0.01; Figure 2). However, significant heterogeneity was observed (*I*^2^ = 81%; *p* < 0.01).

### 3.3. Operative Times in the Primary TKA Cases with and without PJI

**Figure 3:**
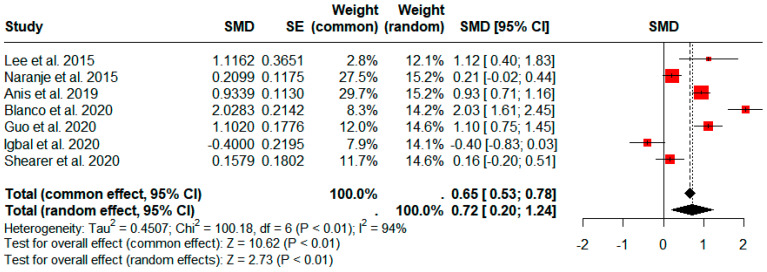
Forest plots showing the operative times of patients with and without periprosthetic joint infection. CI, confidence interval; SE, standard error; SMD, standardized mean difference.

Eight studies comprising 85,194 patients (736 PJIs) provided results for the comparison of operative times between patients with and without PJI [12,19,20,21,32,37,38,39]. The pooled results demonstrated that a significantly longer operative time was associated with PJI (pooled SMD: 0.72; 95% CI: 0.20–1.24; *p* < 0.01; Figure 3). However, significant heterogeneity was observed (*I*^2^ = 94%; *p* < 0.01).

### 3.4. Comparison of the Incidence of PJI between Operative Times of ≥90 and <90 min

**Figure 4:**
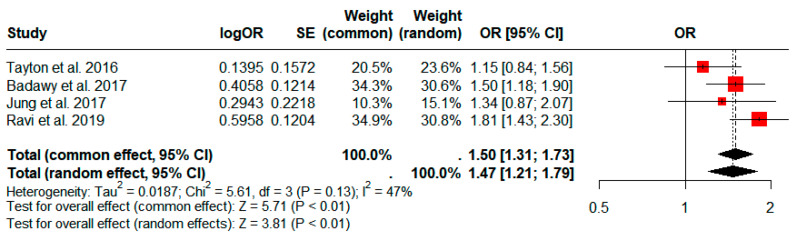
Forest plots comparing the incidence of periprosthetic joint infection in patients with operative times ≥90 min vs. <90 min. CI, confidence interval; OR, odds ratio; SE, Standard error.

Four studies comprising a total of 194,652 patients compared the incidence rate of PJI in patients divided according to a 90 min cutoff for operative time [15,16,34,35]. The group with longer operative times had a significantly higher prevalence of PJI based on our pooled analysis (pooled odds ratio (OR): 1.50; 95% CI: 1.31–1.73; *p* < 0.01; Figure 4).

### 3.5. Comparison of the Incidence of PJI between Operative Times of ≥120 and <120 min

**Figure 5:**
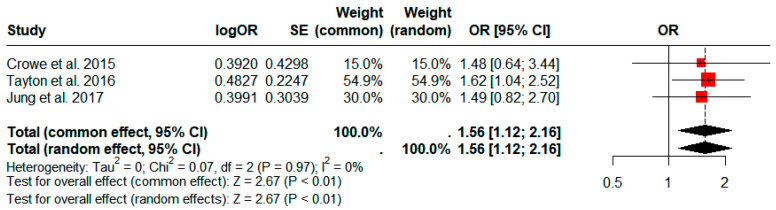
Forest plots comparing the incidence of periprosthetic joint infection between patients with operative times of ≥120 and <120 min. CI, confidence interval; OR, odds ratio; SE, Standard error.

Four studies comprising a total of 89,306 patients compared the incidence rate of PJI in patients divided according to a 120 min cutoff for operative time [22,34,35,38]. The group with longer operative times had a significantly higher prevalence of PJI based on our pooled analysis (pooled OR: 1.56; 95% CI: 1.12–2.16; *p* < 0.01; Figure 5).

### 3.6. Sensitivity Analyses and Publication Bias

Sensitivity analysis was conducted on selected studies to evaluate the impact of individual studies on the overall results. The analysis revealed that the data from one study [39] significantly influenced the pooled results for operative times between TKAs with and without SSIs. Another study [12] similarly impacted the pooled results for operative times between TKAs with and without PJIs, as shown in Figures S1 and S2. As a result, these studies were excluded from their respective meta-analyses. Substantial heterogeneity was observed in the pooled risk of PJI associated with the 120 min operative time cutoff. The sensitivity analysis highlighted the study by Anis et al. [38] as a potential source of this heterogeneity. As a result, this study was excluded from the meta-analysis, as detailed in Figure S3. Furthermore, no evidence of publication bias was detected with the Egger regression-based test (all *p*-values > 0.05).

## 4. Discussion

The present meta-analysis showed that prolonged operative time was associated with a greater risk of PJI in patients undergoing primary TKA. These findings suggest the need for increasing the effort to reduce operative times during TKA. To the best of our knowledge, this is the only published meta-analysis that focuses solely on the incidence of PJI in relation to extended operating times in a primary TKA population.

SSI or PJI after primary TKA requires additional debridement surgery or two-staged revisional arthroplasty, which results in a longer hospital stay, increased morbidity and mortality, and a consequent socioeconomic burden [1,2,3,4,5,7,41]. Efforts to reduce the incidence rates of PJI and SSI after TKA have become increasingly important, and a thorough understanding of modifiable risk factors is essential. Compared with the various patient-related risk factors, such as obesity, diabetes mellitus, and history of operation, operative time is easily assessable and potentially modifiable [4,5,6,7,8].

The exact mechanism by which prolonged operative time increases the incidence rate of infection is multifactorial and poorly understood. With prolonged operative time, open incisions are exposed to microorganisms in the operative environment for a longer period, thus increasing the risk of bacterial contamination [42,43,44,45]. Moreover, a longer operative time predisposes patients to an increased risk of tissue desiccation, which may also increase the probability of contamination [46]. Prolonged operative time is also associated with a longer tourniquet time, which can cause persistent wound hypoxia and may increase susceptibility to infection [47,48]. Furthermore, the tissue concentrations of antibiotics decrease as the operative time increases and may be insufficient if the antibiotics are not re-administered during the surgical procedures [49,50,51,52].

Many potential factors can affect the operative time, including the complexity of the individual case, experience and fatigue of the surgeon, experience of the operating room staff, implant type, and use of cement. Anis et al. demonstrated that younger age, male sex, higher body mass index, low-volume surgeons, and use of antibiotic cements were significantly associated with longer operative times [38]. Furthermore, relationships between minimally invasive approaches, use of computer navigation, and prolonged operative time have been suggested [20,53,54]. Although none of the factors influencing the increase in operative time can be modified, preoperative planning, procedure efficiency, and surgeon education should be optimized to minimize the impact of operating time on the incidence of PJI where possible [55].

Although identifying a cutoff value for a prolonged operative time is important for surgeons to reduce the risk for PJI, no specific value has been defined due to variation among previous studies. Instead, the pooled risk of prolonged operative time was analyzed in accordance with the National Nosocomial Infections Surveillance guidelines by the Centers for Disease Control and Prevention, which recommends a cutoff value of 120 min as the 75th percentile of operative times [24]. However, with advancement in surgical techniques and instruments, a primary TKA is usually performed in around 90 min in expert hands [16,20,36]. As previous studies recommended a shorter cutoff value for prolonged operative times from 80 to 110 min [15,16,34,35,38,39], the pooled risk of prolonged operative time using the 90 min cutoff was further analyzed. The results support that orthopedic surgeons should consider the risk of PJI in cases with prolonged operative times and should aim to reduce the duration where possible.

The present study has both strengths and limitations. One strength is the comprehensive literature search, which included numerous observational studies. Moreover, the association between operative time and PJI was established through quantitative meta-analyses. However, several limitations should be noted: First, only retrospective studies with a low level of evidence were included, leading to some inherent heterogeneity. Despite this, the large cohort sizes and validated reporting systems in national surveillance or registry databases provide reliable data regarding surgical outcomes. Second, significant differences existed in the definitions of operative time, SSI, and PJI, as well as the follow-up durations across studies. This heterogeneity necessitates cautious interpretation of the results. However, the consistency of PJI risk at the 90 and 120 min operative time cutoffs was maintained across studies through sensitivity analysis. Third, operative time is closely associated with tourniquet time, a potential risk factor for increased PJI in prolonged operative cases [47,48]. While some surgeons perform TKA without tourniquets, making the relationship between tourniquet time and PJI clinically important, this study could not analyze the pooled risk of prolonged tourniquet times. Future large cohort studies with controlled confounding factors are needed to conclude the association between operative and tourniquet times and the risk of PJI. Fourth, antibiotic practices can significantly alter the risk of SSIs and PJIs [56,57]. However, the absence of data on antibiotic redosing and extended antibiotic prophylaxis limited our ability to perform a subanalysis on these factors. Furthermore, longer operative times are often correlated with technical problems during surgery, such as higher BMI, which are independent risk factors for PJI. These factors were not comprehensively controlled for in our analysis, highlighting the need for future research to account for such confounders.

## 5. Conclusions

In conclusion, the prolonged operative time was significantly associated with the incidence of PJI and SSI after primary TKA. The risk of PJI was also significantly increased in patients with operative times >90 or 120 min compared to those <90 or 120 min, respectively. Identifying a potentially modifiable risk factor such as operative time is important to achieve better patient outcomes. Given the considerable impact of PJIs and SSIs on patient outcomes and the socioeconomic burden, strategies for infection prevention should incorporate preoperative planning and minimize factors that contribute to a prolonged operative time.

## Figures and Tables

**Table 1:** Baseline characteristics of the included studies.

Author	Publication Year	Study Design	Definition of Infection	Definition of Operative Times	Composition of the Case Group	Number of Patients		Sex (M/F)		Age (y)		Duration of Follow-Up	Controlled Confounding Variables	Threshold of Operative Time (min)
						Case	Control	Case	Control	Case	Control			
Minnema et al. [10] *	2004	Retrospective	CDC criteria	NR	SSI including both superficial and deep infection	22	66	12/10	NR	Median: 68	NR	1 year	Matching for the date of surgery	NR
Namba et al. [12]	2013	Retrospective registry	CDC criteria	NR	Deep surgical site infection	404	55,812	210/194	20,587/35,225	Mean: 66.5	Mean: 67.4	3 months	None	NR
Crowe et al. [22]	2015	Retrospective	CDC criteria	NR	Deep surgical site infection	26	3393	NR	NR	NR	NR	NR	None	114
Lee et al. [33]	2015	Retrospective	Criteria of the Musculoskeletal Infection Society	NR	PJI	8	192	4/4	53/139	Mean: 62.1	Mean: 69.2	2 years	Matching for the year of surgery	NR
Naranje et al. [20]	2015	Retrospective registry	Reoperation for PJI	Time from skin incision to dressing placement	Reoperation for PJI	73	9900	26/47	NR	Mean: 66.2	NR	NR	None	NR
Tayton et al. [34]	2016	Retrospective registry	Reoperation for PJI	NR	Reoperation for PJI	179	64,387	112/67	31,068/33,319	≥65 years (61.5%)	≥65 years (64.7%)	Minimum 1 year	None	90 and 120
Badawy et al. [15]	2017	Retrospective registry	Reoperation for PJI	Time from skin incision to skin closure	Reoperation for PJI	311	27,951	10,186/18,076 (whole cohort)		≥70 years (48.0% of the whole cohort)		Minimum 1 year	None	90
Jung et al. [35]	2017	Retrospective registry	CDC criteria	NR	Deep surgical site infection	44	9437	31/13	4348/5085	≥65 years (52.3%)	≥65 years (68.4%)	3 months	None	90 and 120
George et al. [36]	2018	Retrospective registry	SSI including superficial and deep infection	Time from skin incision to skin closure	SSI including both superficial and deep infection	1121	139,078	52,855/87,344 (whole cohort)		≥65 years (59.4% of the whole cohort)		1 month	None	NR
Hao et al. [17]	2018	Retrospective	Deep surgical site infection	NR	Deep surgical site infection	16	1145	7/9	221/924	Mean 62.8	Mean 64.4	Mean: 1.6 year	None	NR
Teo et al. [37]	2018	Retrospective	Criteria of the Musculoskeletal Infection Society	Time from skin incision to skin closure	SSI including both superficial infection and PJI	10	895	2/8	192/703	Mean 65.4	Mean 65.9	2 years	None	NR
Anis et al. [38]	2019	Retrospective	Reoperation for infection	Time from skin incision to skin closure	PJI	79	11,761	NR	NR	NR	NR	Mean: 2 years	None	85,102 and 121
Ravi et al. [16]	2019	Retrospective registry	Reoperation for PJI	Time from the entry to exit into the operating room	Reoperation for PJI	839	91,504	NR	NR	NR	NR	1 year	Age, sex, obesity, primary surgeon, hospital, type of anesthesia	100
Blanco et al. [39]	2020	Retrospective	Criteria of the Musculoskeletal Infection Society	NR	PJI	66	66	20/46	28/38	Mean 70.1	Mean 72.4	NR	Matching for the year of surgery	90
Guo et al. [40]	2020	Retrospective	Criteria of the Musculoskeletal Infection Society	NR	PJI	54	108	NR	NR	Mean 66.8	Mean 67.2	1 year	Matching for the year of surgery	NR
Iqbal et al. [19]	2020	Retrospective	CDC criteria	NR	PJI	48	4221	28/200	1427/2794	Mean 61.2	Mean 61.5	NR	None	NR
Shearer et al. [21]	2020	Retrospective	Criteria of the Musculoskeletal Infection Society	Time from skin incision to skin closure	PJI	31	4114	21/10	2070/2644	Mean 66.8	Mean 68.3	1 year	None	NR

M, male; F, female; y, year(s); NR, not reported; CDC, the Centers for Disease Control and Prevention; SSI, surgical site infection, PJI, periprosthetic joint infection. * Reference number.

**Table 2:** Quality assessment of the included studies.

Author	Publication Year	Selection				Comparability	Outcomes			Overall
		Representativeness of the Exposed Cohort	Selection of the Nonexposed Cohort	Ascertainment of Exposure	Demonstration That the Outcome of Interest Was Not Present at the Start of the Study		Assessment of Outcomes	Sufficient Follow-Up	Adequacy of Follow-Up	
Minnema et al. [10] *	2004	★	★	★	★	★	★	★	★	8
Namba et al. [12]	2013	★	★	★	★	☆	★	☆	★	6
Crowe et al. [22]	2015	★	★	★	★	☆	★	☆	☆	5
Lee et al. [33]	2015	★	★	★	★	★	★	★	★	8
Naranje et al. [20]	2015	★	★	★	★	☆	★	☆	☆	5
Tayton et al. [34]	2016	★	★	★	★	☆	★	★	★	7
Badawy et al. [15]	2017	★	★	★	★	☆	★	★	★	7
Jung et al. [35]	2017	★	★	★	★	☆	★	☆	★	6
George et al. [36]	2018	★	★	★	★	☆	★	☆	★	6
Hao et al. [17]	2018	★	★	★	★	☆	★	★	★	7
Teo et al. [37]	2018	★	★	★	★	☆	★	★	★	7
Anis et al. [38]	2019	★	★	★	★	☆	★	★	★	7
Ravi et al. [16]	2019	★	★	★	★	★★	★	★	★	9
Blanco et al. [39]	2020	★	★	★	★	★	★	☆	☆	6
Guo et al. [40]	2020	★	★	★	★	★	★	★	★	8
Iqbal et al. [19]	2020	★	★	★	★	☆	★	☆	☆	5
Shearer et al. [21]	2020	★	★	★	★	☆	★	★	★	7

☆, No star; ★, One star, ★★, Two stars. * Reference number.

## Data Availability

The data presented in this study are available on request from the corresponding author.

## Supplementary Materials

The following supporting information can be downloaded at: https://www.mdpi.com/article/10.3390/jcm13113046/s1, Figure S1: Sensitivity analysis for operative times between TKAs with and without SSI; Figure S2: Sensitivity analysis for operative times between TKAs with and without PJI; Figure S3: Sensitivity analysis for the risk of PJI according to a 120-min cutoff for operative time.
