# Supplementary material for: Greater Risk of Periprosthetic Joint Infection Associated with Prolonged Operative Time in Primary Total Knee Arthroplasty: Meta-Analysis of 427,361 Patients
Source: J Clin Med. 2024 May 22;13(11):3046. doi: 10.3390/jcm13113046 (PMC11172656; doi:10.3390/jcm13113046)
Supplement: Supplementary file 1 [file jcm-13-03046-s001.zip › jcm-3002375-supplementary.pdf]

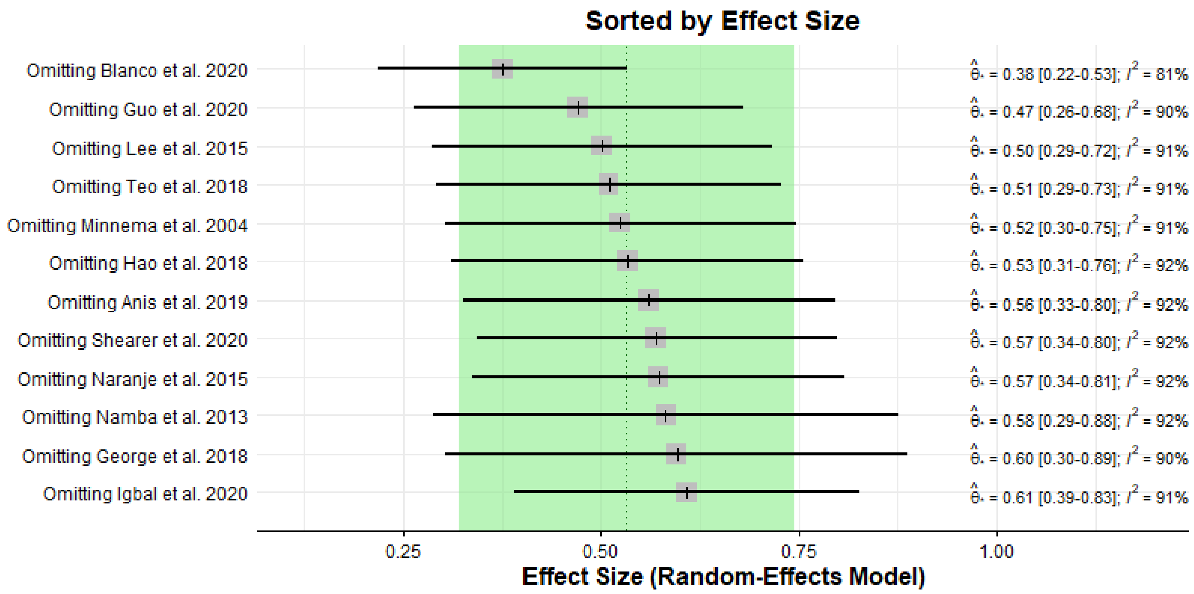

Supplementary Figure S1. Sensitivity analysis for operative times between TKAs with and without SSI

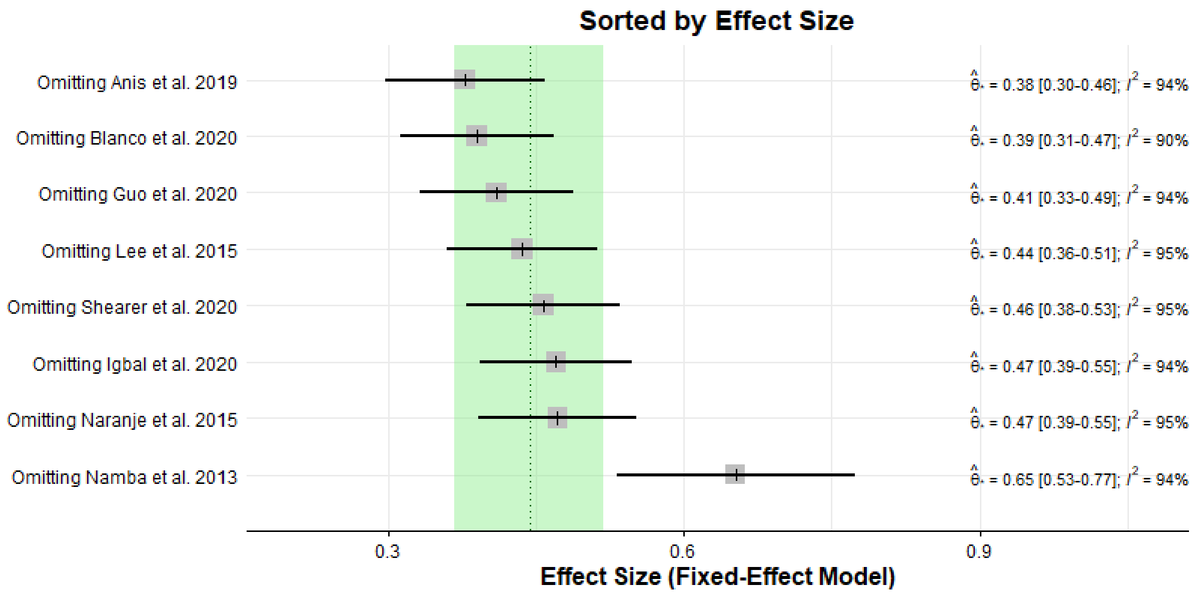

Supplementary Figure S2. Sensitivity analysis for operative times between TKAs with and without PJI

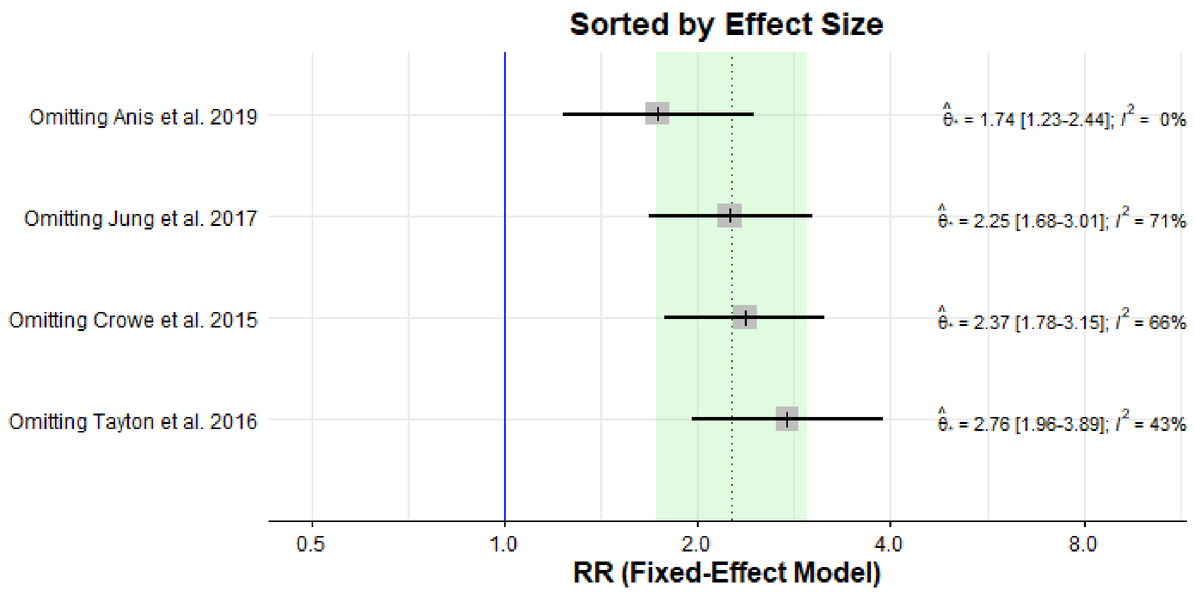

Supplementary Figure S3. Sensitivity analysis for the risk of PJI according to a 120-min cutoff for operative time
